# Analysis of the Effect of Different Physical Exercise Protocols on Depression in Adults: Systematic Review and Meta-analysis of Randomized Controlled Trials

**DOI:** 10.1177/19417381231210286

**Published:** 2023-11-22

**Authors:** Érica M. Correia, Diogo Monteiro, Teresa Bento, Filipe Rodrigues, Luís Cid, Anabela Vitorino, Nuno Figueiredo, Diogo S. Teixeira, Nuno Couto

**Affiliations:** Sport Sciences School of Rio Maior, Polytechnic of Santarém [ESDRM-IPSantarém], Rio Maior, Portugal; ESECS - Polytechnic of Leiria, Leiria, Portugal and Research Center in Sports Sciences, Health Sciences and Human Development [CIDESD], Vila Real, Portugal; Sport Sciences School of Rio Maior, Polytechnic of Santarém [ESDRM-IPSantarém], Rio Maior, Portugal; and Research Center in Sports Sciences, Health Sciences and Human Development [CIDESD], Vila Real, Portugal; ESECS - Polytechnic of Leiria, Leiria, Portugal and Life Quality Research Center [CIEQV], Rio Maior, Portugal; Sport Sciences School of Rio Maior, Polytechnic of Santarém [ESDRM-IPSantarém], Rio Maior, Portugal; and Research Center in Sports Sciences, Health Sciences and Human Development [CIDESD], Vila Real, Portugal; Sport Sciences School of Rio Maior, Polytechnic of Santarém [ESDRM-IPSantarém], Rio Maior, Portugal; Research Center in Sports Sciences, Health Sciences and Human Development [CIDESD], Vila Real, Portugal, and Life Quality Research Center [CIEQV], Rio Maior, Portugal; ESECS - Polytechnic of Leiria, Leiria, Portugal; Faculty of Physical Education and Sport, Lusófona University [ULHT/FEFD], Lisbon, Portugal, and Research Center in Physical Education, Sport, Exercise and Health [CIDEFES], Lisbon, Portugal; Sport Sciences School of Rio Maior, Polytechnic of Santarém [ESDRM-IPSantarém], Rio Maior, Portugal and Research Center in Sports Sciences, Health Sciences and Human Development [CIDESD], Vila Real, Portugal

**Keywords:** adult, depression, physical exercise, treatment

## Abstract

**Context:**

Physical exercise (PE) is an effective treatment for depression, alone or as an adjunct.

**Objective:**

There is a lack of indicators regarding the frequency, intensity, duration, and type of physical exercise (PE). This study aims to synthesize and analyze the dose-effect of different PE protocols in adult subjects in the treatment of depression, based on the analysis of randomized controlled trials (RCTs).

**Data Sources:**

The search was conducted using Web of Science, PubMed, and Cochrane Library electronic databases.

**Study Selection:**

Studies with an exercise-based intervention published by December 31, 2021 were identified. RCTs and meta-analyses involving adults with depression were also included; 10 studies were selected, including a total of 956 subjects.

**Study Design:**

Systematic review and meta-analysis.

**Level of Evidence:**

Level 1.

**Results:**

Effect sizes were summarized using standardized mean differences (95% confidence interval) by effected randomized models. The results reinforce that exercise appears to be beneficial in improving depression among adults aged 18 to 65 years. Interventions lasting above 150 minutes per week of moderate intensity and group interventions seem to have a more significant effect on reducing depression. Studies have revealed that aerobic exercise, compared with resistance or flexibility, has a more positive effect on depression.

**Conclusion:**

PE can be a way to reduce depression and can be used as a possible adjunctive tool for pharmacological and/or alternative treatments. Considering the findings of this study, it is important that health professionals (eg, exercise physiologists, physicians, nurses, psychologists) promote the practice of PE as a complementary alternative and act early to prevent the worsening of depression.

**PROSPERO Registration Number:**

CRD42020188909

Worldwide, about 1 in 6 people suffers from some type of mental disorder during their lifetime.^24^ According to the World Health Organization,^51^ depressive disorders are the third main cause of global disease burden (the first in developed countries), and are expected to be the first, worldwide, by the year of 2030. Depression is the main cause of incapacity in adults worldwide, with nearly 300 million people suffering from this mental health condition every year.^25^

Depression is a common mental health disorder and may have a great impact in individual well-being and in daily functional capacity.^25^ Depression is consistently classified among the 10 main causes of incapacity all over the world,^50^ displaying a set of affective (eg, sadness), cognitive (eg, feelings of guilt), and somatic (eg, loss of appetite) symptoms.^2^ In its most severe form, depression can lead to suicide.^9,30^

The median age of onset of major depressive disorder is 40 years, with 50% of all patients having onset between 20 and 50 years.^3,4^ Depression is estimated to cause 200 million lost workdays each year at a cost of $17 billion to $44 billion to employers, according to the Centers for Disease Control and Prevention (CDC).^20^

Treatment of depression (pharmacological or otherwise) relies on some variables, namely severity of symptoms (ie, light, moderate, or severe symptoms), triggering factors (ie, severe depressive symptoms, prolonged duration of the depressive episode, and the presence of clinical and/or psychiatric comorbidities), type of symptoms present (ie, depressive humor or lack of interest/pleasure), available resources in the context of care (easy-to-use support materials), patient preferences, and familiarity of the professional with the method (maintenance dose).^6^

Regarding treatment of depression, it is important to point out complementary therapies that can be used; among those, physical exercise (PE) is increasingly recognized as an effective means of treatment and/or as an alternative to treat depression.^18,19,35,38^

PE can decrease depressive symptoms and increase the quality of life of people suffering from depression.^43–45^ PE can also have an impact on well-being indicators in this population^32^ and can influence depressive symptoms through a variety of psychosocial and biological mechanisms, such as the stimulation of neuroplasticity, the reduction of inflammatory cytokines interleukin (IL)-6, tumour necrosis factor alpha (TNFα), and IL-1β; the promotion of self-esteem^26^; increased release of hormones such as catecholamines, adrenocorticotropic hormone, vasopressin, B-endorphin, dopamine, serotonin; and by the activation of specific receptors (ie, β-adrenergic), as well as by the decrease of blood viscosity, providing an analgesic, tranquilizing, and relaxing effect after effort.^33,34^ Although PE variables vary across analyzed studies in previous reviews, evidence suggests that even low PE doses can protect against depression.^7,23,47^

The effectiveness of structured PE programs in the reduction of depression is well established.^27,41^ The success of these interventions encourages further research into the ways in which exercise can be used to augment existing treatments. Several meta-analyses regarding the intervention of PE in depression have verified a moderate-to-high antidepressant effect of PE.^44,47^ Hence, the Canadian Network for Mood and Anxiety Treatments has revised the treatment guidelines for depression,^39^ and now recommends PE as a primary intervention for light-to-moderate depression, and as a secondary therapy for moderate-to-severe depression.^39^ Similar recommendations are also present in the guidelines of the National Institute for Health and Care Excellence for the treatment of depression.^37^ With moderate-to-high effect sizes, the antidepressants properties of PE have also turned out to be comparable with those of psychotherapy and antidepressants.^29,38,40,47^ Consequently, the guidelines of the National Institute for Health and Care Excellence for the treatment of depression include the recommendation to implement PE as a therapeutic method to complement the standard treatment for depression.^36^

PE continues to be an active area of research, raising questions about dose response and the best type of exercise for various depressed patients. Given the high levels of insufficient exercise in the global population,^28^ further exploration of the psychological correlates of “movement as medicine” for the adult population has great public health significance. However, this aspect does not seem to receive proper attention, and its use in clinical practice is not equitable compared with dominant strategies such as pharmacotherapy and psychotherapy.^15^

Although several randomized controlled trials (RCTs) suggest that exercise is an effective treatment for depression, either alone or in combination, recent results from systematic reviews point to few methodologically robust studies and small-to-moderate effect sizes, including few PE types, PE duration, and PE frequency, making dose-response relationships elusive.^11^ Thus, the present study aims to synthesize and analyze the dose-effect of different PE protocols in adult subjects in the treatment of depression. Specifically, this study aims to determine the recommended dose of PE (frequency, intensity, duration, and type) on depressive symptomatology.

## Methods

### Search Strategy

The literature search was carried out on the Web of Science, PubMed, and Cochrane Library using the PICOS (population, intervention, comparison, outcome, and study design) search strategy (Table 1). Literature published until December 31, 2021, was included. Two reviewers independently performed the search and evaluated the eligibility of each article. Doubts regarding the inclusion or exclusion of studies were resolved through the discussion between the 2 independent researchers. To assure the quality of the study, the present systematic review and meta-analysis was prepared according to the Cochrane systematic review guidelines and to the preferred reporting items for systematic review and meta-analysis protocols (PRISMA-P, 2020).^42^

**Table 1.** PICO: categories and keywords used for study identification

Categories	Keywords
Population	Adults; women; men; depressive; depression
Intervention	Intervention; program; treatment; physical exercise
Comparison	PE program effect; control group and intervention group; duration, frequency, and exercise type
Outcome	Depression; well-being; physical well-being; social well-being; psychological well-being; quality of life; physical fitness
Study design	RCT

PICO, population, intervention, comparison, outcome; RCT, randomized controlled trial.

The keywords used in the database search were “depression,” “adults,” “interventions,” “physical exercise,” “exercise,” and “programs”, using the search expression (depression) AND (exercise) OR (physical exercise) AND (intervention) OR (programs) OR (RCT).

The survey was conducted without any time period, and a specification was considered as to the document type and language, selecting scientific papers written in English. This review was registered in the PROSPERO international prospective register of systematic reviews (registration number: CRD42020188909).

### Eligibility Criteria

The inclusion criteria for this systematic review were as follows: (1) RCTs (parallel group or cluster randomized); (2) a study population aged between 18 and 65 years; (3) a diagnosis of depression following the criteria established by The Diagnostic and Statistical Manual of Mental Disorders, Fifth Edition (DSM-5); (4) an intervention protocol; (5) destined to assess different doses and effects of a PE protocol; and (6) assessment of ≥1 of the symptoms present in patients with depressive symptomatology. The following documents were excluded from this systematic review: (1) literature reviews of any sort; (2) thesis and dissertations (master and doctorate); (3) articles composed by multidisciplinary/interdisciplinary interventions or with clinical diseases in comorbidity; (4) educational or cognitive-behavioral therapies; (5) articles presenting subjects with >1 medical diagnosis besides depression; and (6) research without a control group.

### Study Selection

Articles were selected according to PRISMA recommendations. All studies identified in the literature search were selected by at least 2 independent reviewers. Endnote version 20.2 (EndNote, Clarivate Analytics) was used to gather articles from different databases as well as to exclude duplicates.

First, the titles and abstracts of the articles from the initial search were selected according to the eligibility criteria mentioned above. Second, full articles were examined in detail and selected for eligibility. Then, the reference lists of all the articles were screened to identify any relevant publications missed in the database search. In case of disagreement between reviewers, another element was included to achieve a final decision.

### Data Extraction

After selecting the studies to be included in the present study, data related to the study selection process were extracted, as well as publication information, study design, study population (ie, participant characteristics), intervention methods (content of the intervention toward control and intervention groups including duration, frequency, intensity, and type of PE), theoretical frameworks, measurement instruments, and outcomes of the intervention (ie, results on the effectiveness on depression). The details mentioned were then synthesized and inserted into a flowchart. As a reference, we proceeded as recommended by PRISMA.^42^ Authors who did not provide the necessary data for the meta-analysis were contacted by email to obtain the information. For those who did not respond, the necessary information was acquired through old systematic reviews.

### Risk of Bias Assessment

The risk of bias analysis was addressed separately by 2 reviewers, according to the methods recommended by the Cochrane Collaboration. Any differences between researchers were resolved by a third reviewer. Through the Cochrane Collaboration tool, the following criteria were considered: (1) sequence generation (selection bias); (2) allocation sequence concealment (selection bias); (3) blinding of participants and personnel (performance bias); (4) blinding of outcome assessment (detection bias); (5) incomplete outcome data (attrition bias); (6) selective outcome reporting (reporting bias); and (7) other potential sources of bias. For these criteria, the following classifications were used: “low risk,” “high risk,” or “unclear risk,” with the latter indicating either lack of information or uncertainty regarding a potential bias. To create graphics regarding the risk of bias, the Review Manager (RevMan, The Nordic Cochrane Center), Version 5.4 software was used.^41^

### Data Analysis

The meta-analysis was conducted with the RevMan Version 5.4 to determine the effects of different types of PE effects and the frequency, intensity, duration, and type on reducing depression.^41^ The standard mean difference of depression measurements pre- and postintervention were calculated. The standard deviation, when not presented in the studies, was estimated using procedures recommended in the Cochrane handbook by Higgins et al.^22^ Only articles including this information were included in the meta-analysis. The random effects model for an inverse variation was used to calculate the mean difference and the 95% confidence interval).

The *I*² statistic was used to measure the heterogeneity of the RCTs, considering the classification established by Higgins et al^22^ (*I*^2^ < 25% low; *I*^2^ = 50% to 75% moderate; *I*^2^ > 75% high). To classify the magnitude of the effect size, the Cohen’s category was selected (*d* values between 0.2 and 0.5 represent a small effect size; between 0.5 and 0.8 a moderate effect size; >0.8 a large effect size). In the present meta-analysis, negative effect size values favor the intervention of PE while positive values favor the control group.

## Results

**Figure 1.**
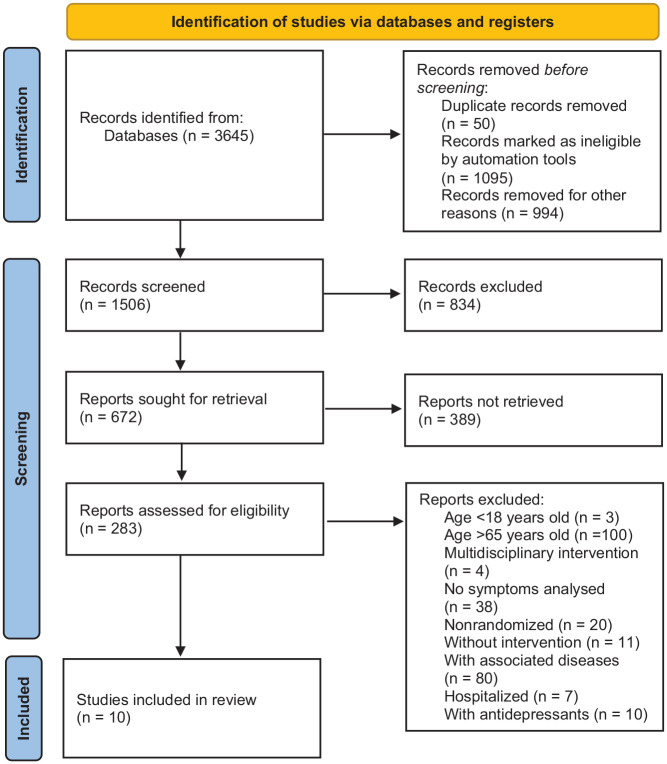
Flowchart of the selection and steps to We send you a new figure attached without symbols.

The literature search identified 3645 publications, which were reduced to 1506 after refining by areas of interest. After analyzing the titles/abstracts, 1223 articles were excluded. Therefore, 283 publications were obtained for detailed analysis, and the reasons for exclusion were population, n = 200; intervention, n = 4; outcome, n = 38; and type of study, n = 31 (Figure 1).

Appendix Table A1, available in the online version of this article, shows the characteristics of the included studies, specifically the participants, duration, intervention, and main results. Overall, 956 patients with depression were included in the studies, and 505 were allocated to a PE program. The mean age of all subjects was 40.04 years (range, 18-69 years), and the samples were composed mainly of female (56%). For measuring depression, the included studies used the Beck Depression Inventory (BDI-II) (n = 7), the Montegomery-Asberg Depression Rating Scale (MADRS) (n = 3), the Patient Health Questionnaire 9 (PHQ-9) (n = 1), or the Depression, Anxiety and Stress Scale (DASS-21) (n = 2).

Among the different variables evaluated, the PE effect was the parameter analyzed most, as described by the different types and intensity of PE. The minimum intervention period presented by 1 of the articles was 3 weeks,^21^ whereas the maximum period lasted 16 weeks.^8,10^ The frequency and duration of each session varied between 2 and 5 times per week,^14,17,21,43^ The shortest session, in 1 study,^21^ was 15 minutes and the maximum session time varied between 60 and 75 minutes.^14,43^

Regarding the PE protocols used, 2 studies evaluated a combined protocol consisting of flexibility, aerobic, and resistance training,^32,43^ 1 study evaluated a combined protocol of aerobic exercise and flexibility,^47^ and 5 studies focused on aerobic exercise.^8,14,21,48,50^

Concerning intensity, some studies created a protocol with progression of PE intensity every week,^8,21,32,43,46,50^ whereas others made the adjustment in each session, depending on participant autonomy.^8,14,17,47^

Some results were evaluated by >1 article, and the PE protocols had a greater impact on the improvement of depression among the evaluated population, namely reduction of depressive symptoms, improvement of cardiovascular fitness, increase of body flexibility, and improvement of quality of life.

**Figure 2.**
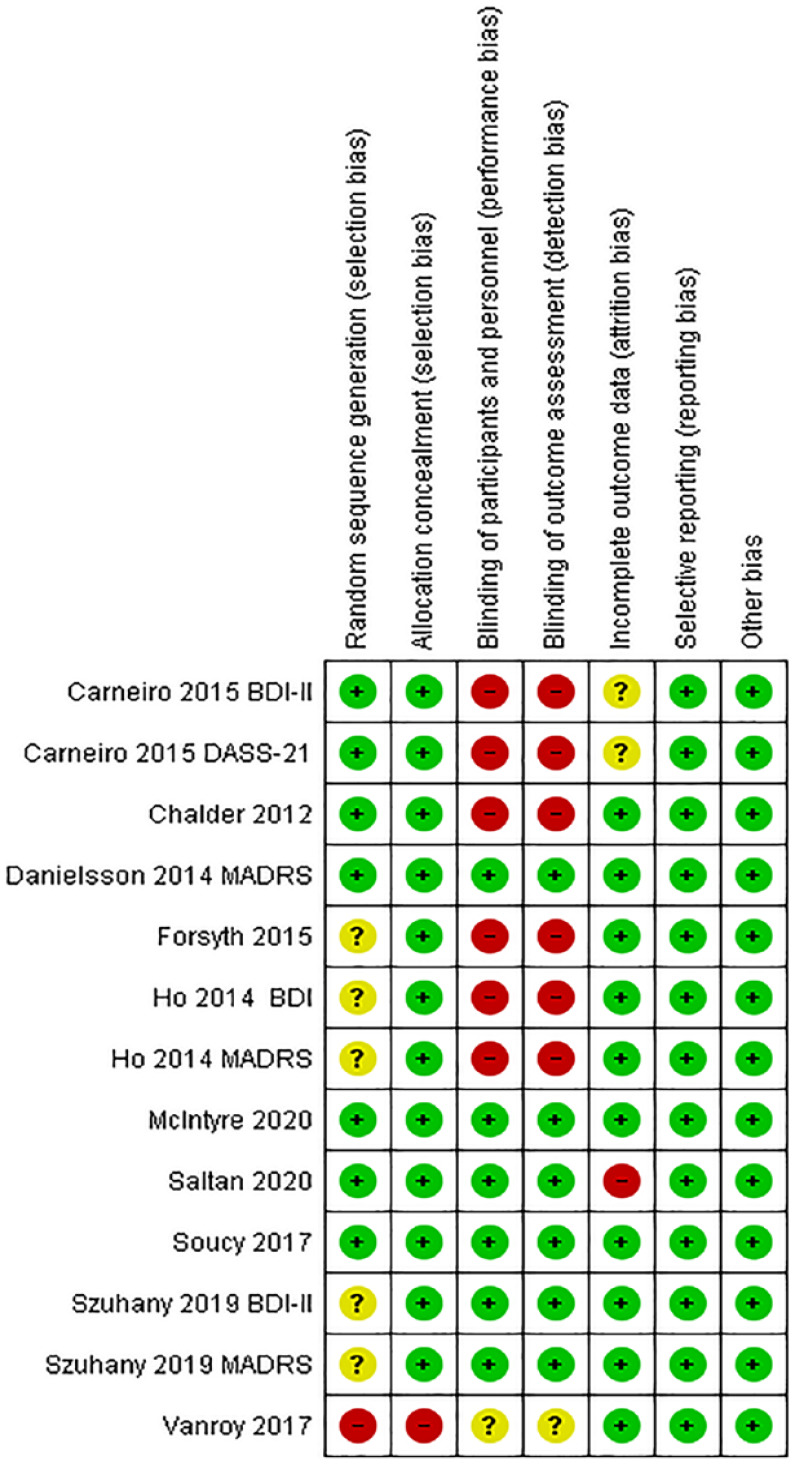
Summary of the risk of bias for each item for each article included in the study (+ low 4 risk, ? unclear risk, - high risk).

Regarding the risk of bias, although all the articles included were RCTs, 6 of the studies demonstrated a low risk of bias for generation of random sequence,^8,10,14,32,43,48^ and no article displayed uncertain risk of bias for attribution concealment. Only 4 of the articles had a low risk of bias for blinding of participants and professionals (see Figure 2).^32,14,43,47^

**Figure 3.**
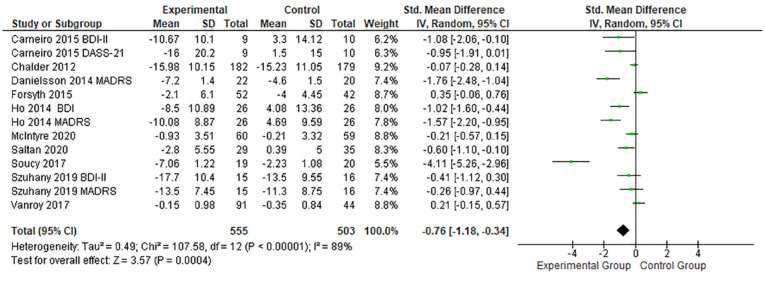
Forest plots showing the effects of PE on depression. IV, inverse variance; PE, physical exercise; SMD, standard mean difference.

The results shown in Figure 3 provide a comparison between the experimental and the control groups, to analyze the effect of PE on depression. All the PE interventions displayed a beneficial effect regarding the reduction of depression (standardized mean difference [SMD], -0.76; 95% CI, -1.18 to -0.34; *P* < 0.01).

**Figure 4.**
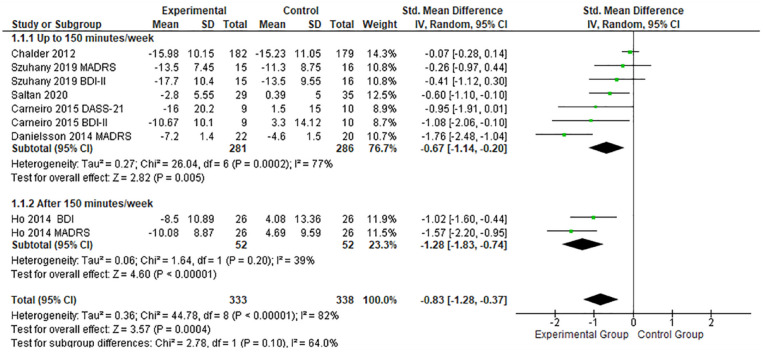
Forest plots showing the effects of PE frequency (minutes per week) on depression. IV, inverse variance; PE, physical exercise; SMD, standard mean difference.

Considering the outcome effect of PE as one of the most relevant variables for the person with depression, a deeper analysis regarding session time and intervention period was conducted (Figure 4). Studies with interventions above 150 minutes per week showed a high effect size (SMD, -1.28; 95% CI, -1.83 to -0.74; *P* < 0.01),^21^ compared with interventions below 150 minutes per week,^8,10,14,43,47^ which, on the other hand, had a greater heterogeneity index.

**Figure 5.**
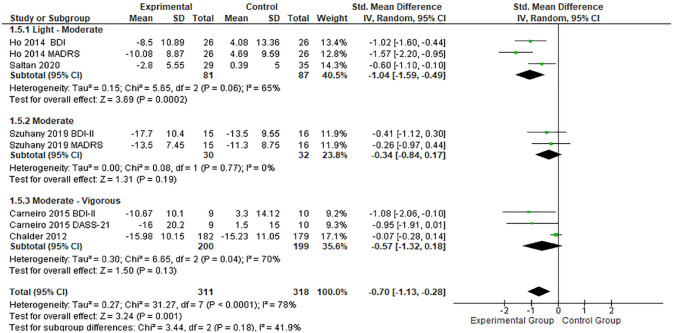
Forest plots showing the effects of PE intensity on depression. IV, inverse variance; PE, physical exercise; SMD, standard mean difference.

Regarding PE intensity, studies with mild-to-moderate intensity interventions showed greater effects (SMD, -1.04; 95% CI, -1.59 to -0.49; *P* < 0.01),^21,43^ compared with moderate intensity interventions (SMD, -0.34; 95% CI, -0.84 to 0.17; *P* < 0.01) as shown in Figure 5.

**Figure 6.**
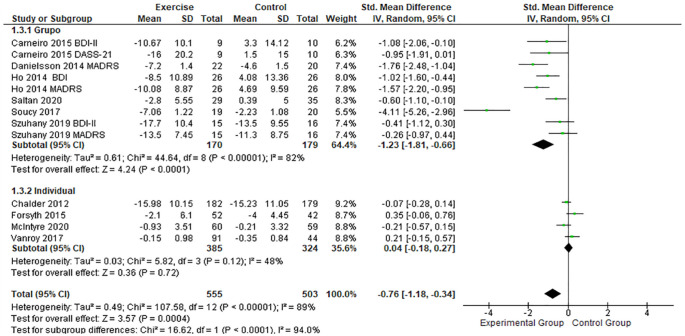
Forest plots showing the effects of PE in group and individual sessions on depression. IV, inverse variance; PE, physical exercise; SMD, standard mean difference.

Comparing the effect of exercising in group and individually (Figure 6), it was possible to identify that both situations have a positive effect on depression. However, group sessions have a more significant positive effect (SMD, -1.23; 95% CI, -1.81 to -0.66; *P* < 0.01) compared with individual PE sessions (SMD, 0.04; 95% CI, -0.18 to 0.27; *P* < 0.01).^8,14,21,43,46,47^

**Figure 7.**
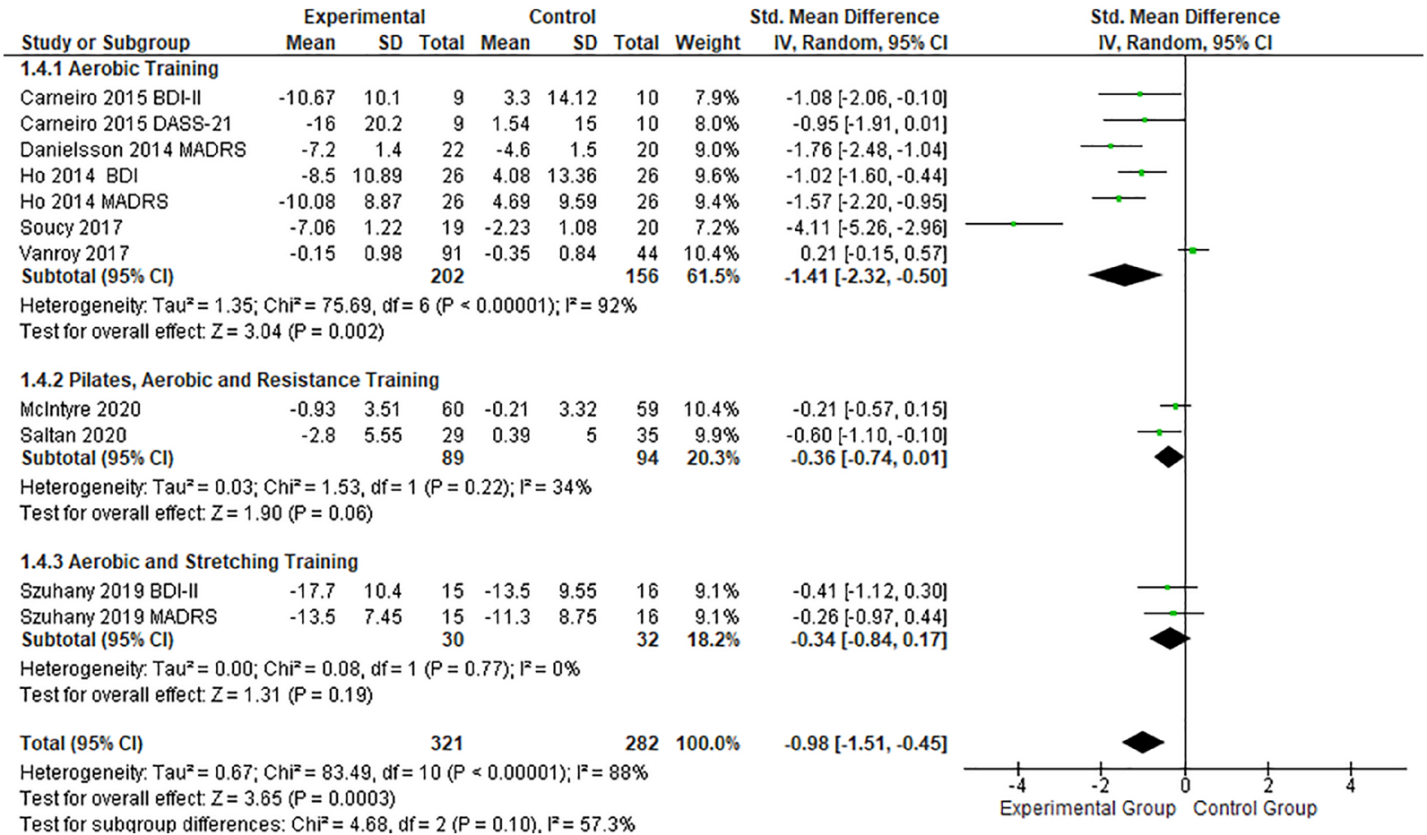
Forest plots showing the effects of different types of PE on depression. IV, inverse variance; PE, physical exercise; SMD, standard mean difference.

In subgroup analysis (Figure 7), aerobic training displayed a large positive effect (SMD, -1.41; 95% CI, -2.32 to -0.50; *P* < 0.01),^8,14,21,46,50^ whereas interventions including combined training (aerobic and flexibility, aerobic and resistance) had a moderate effect (SMD, -0.36; 95% CI, -0.74 to 0.01; *P* < 0.01).^32,43^

## Discussion

The aim of the present systematic review with meta-analysis was to systematically examine the recommended dose of PE in the treatment of depression in adults. The present meta-analysis allowed us to better understand the effects of PE on the reduction of depressive symptomatology. The different types, intensities, and frequencies of PE were able to improve and to reduce the impact of depression on the social, physical, and psychological well-being of persons with depression. Almost all studies had a beneficial effect with respect to the depression parameter. In line with these results, the studies have shown that performing PE programs is beneficial to this population, and the early introduction of exercise in the treatment of depression may have a synergistic effect.^8,10,14,17,21,32,43,46,47,50^

To facilitate the analysis of the intervention, and its frequency, the articles were divided into 2 categories: (1) <150 minutes per week PE and (2) >150 minutes per week PE.

All PE protocols were performed with a maximum time of 75 minutes, with the study conducted by Ho et al^21^ displaying the smallest time of aerobic intervention, consisting of 40 minutes, with significant results. By focusing on combined programs,^32,43^ the studies showed a moderate effect on the impact of depression. On the other hand, protocols with PE performed autonomously by patients.^10,17,32,50^ despite including aerobic exercise, showed a small, but significant, effect size. The analyzed literature suggests that these protocols may reduce depression.

Programs with greater weekly session time (>150 minutes) seem to be more beneficial in the reduction of depressive symptomatology.^21^ This supports existing literature following the PE recommendations for adults,^1^ in which >150 minutes per week of aerobic exercise can produce beneficial effects on physical and mental health. To promote positive effects, we suggest that an intervention protocol needs to have a frequency of >150 minutes per week, consisting of 3 weekly sessions, for a period of between 3 and 8 weeks. As far as intensity is concerned, this meta-analysis showed that moderate intensity interventions had a more positive effect compared with a vigorous intensity intervention, as well as group interventions compared with individual training sessions.

In this review, several studies reported the benefits of aerobic exercise in reducing depressive signs and depression in the population. Seven articles aimed to evaluate the effectiveness of PE in the treatment of depression,^8,10,14,17,32,46,50^ but only 2 studies assessed the effectiveness of aerobic training on depression,^21,47^ and 1 study analyzed the effect of flexibility on the same condition.^43^

Analyzing the results obtained from the current review, 2 programs consisting of aerobic interventions,^8,21,14,48,50^ and 3 studies of combined exercise,^32,43^ displayed favorable effects on depression. However, aerobic exercise displayed a greater effect. The literature presents a set of different training methods (ie, aerobic, resistance, flexibility, and combined interventions), but a common factor among the studies is the use of aerobic exercises. Aerobic exercise produces a positive effect on physical performance, substantially reducing depression.^10^ It is now well established that aerobic PE exerts a protective effect against disease,^12^ not only of cardiovascular nature^5^ but also against all-cause mortality.^31,49^ The combined protocols displayed a moderate-to-high effect. However, aerobic only protocols had greater effect size. Consequently, the high heterogeneity reveals a tendency toward the need for more investigations regarding PE in adults with depressive symptomatology.

Several meta-analyses and systematic reviews have shown that PE may be as effective as psychotherapy and pharmacotherapy in the treatment of mild-to-moderate depression.^13,14^ Evans-Lacko et al^16^ revealed through a meta-analysis that PE had a significantly large antidepressant effect and, as such, the authors described PE as an evidence-based treatment for depression. This is aligned with the recommendations contemplated by the Canadian Network for Mood and Anxiety Treatments regarding the treatment of depression in adults.^39^ PE received Level 1 evidence and was recommended as first-line monotherapy for mild-to-moderate depression.^40^

However, every study chose to primarily evaluate the effectiveness of PE and alternative nonpharmacological interventions for this population. Although PE programs are recommended, there are still many gaps that may affect the results, such as programs performed autonomously by persons themselves. Therefore, more research is needed.

## Limitations and Perspectives for Future Studies

Despite the important contributions of this systematic review with meta-analysis for depression, especially regarding PE interventions and their effect on persons diagnosed with the referred condition, some limitations must be mentioned. Limitations of the studies may have influenced the results, such as lack of information regarding the training program, namely load progression (ie, intensity and volume) during the intervention period, or incomplete information, making it impossible to analyze some variables. For instance, not all articles evaluated in this analysis described complete data about the duration of the sessions and/or their weekly frequency. Because of the difference in the methodological quality displayed by the studies included in the present systematic review, as well as the risk of bias presented in the protocols of some of the articles, the results obtained must be considered and analyzed carefully.

## Conclusion

The present study aimed to synthesize and analyze the dose-effect of different PE protocols in adult subjects in the treatment of depression. Specifically, this study aimed to determine the recommended dose of PE (ie, frequency, intensity, duration, and type) in depressive symptomatology.

The results reinforce the notion that PE seems to be beneficial for the improvement of depression among adults aged between 18 and 65 years.

Therefore, intervention protocols on depression are appropriate and can be used as a possible adjuvant treatment tool to pharmacological and/or alternative treatment. However, interventions with aerobic exercises displayed more positive and significant effects compared with interventions with combined exercises, namely resistance, aerobic, and flexibility. Yet, these studies still showed a moderate effect in the mentioned population. Nevertheless, the present study stands out and differs from previous studies, since it was possible to identify that exercise programs with a duration of 3 to 8 weeks must be applied as a mean to reduce depression, and each session should last between 40 and 60 minutes. That is, the frequency should be >150 minutes per week, at a frequency of approximately 2 to 3 times per week. Further, each training program should be performed in groups, starting with light intensity, and gradually increasing to moderate intensity through time.

We conclude that PE can reduce depression and can be a possible adjunctive tool for pharmacological and/or alternative treatments. Considering the findings of this study, it is important that health professionals (eg, exercise physiologists, physicians, nurses, psychologists) promote PE as a complementary alternative and act early to prevent the worsening of depression.

## Supplemental Material

sj-docx-1-sph-10.1177_19417381231210286 – Supplemental material for Analysis of the Effect of Different Physical Exercise Protocols on Depression in Adults: Systematic Review and Meta-analysis of Randomized Controlled Trials Supplemental material, sj-docx-1-sph-10.1177_19417381231210286 for Analysis of the Effect of Different Physical Exercise Protocols on Depression in Adults: Systematic Review and Meta-analysis of Randomized Controlled Trials by Érica M. Correia, Diogo Monteiro, Teresa Bento, Filipe Rodrigues, Luís Cid, Anabela Vitorino, Nuno Figueiredo, Diogo S. Teixeira and Nuno Couto in Sports Health
