# Supplementary material for: Analysis of the Effect of Different Physical Exercise Protocols on Depression in Adults: Systematic Review and Meta-analysis of Randomized Controlled Trials
Source: Sports Health. 2023 Nov 22;16(2):285–94. doi: 10.1177/19417381231210286 (PMC10916777; doi:10.1177/19417381231210286)
Supplement: sj-docx-1-sph-10.1177_19417381231210286 – Supplemental material for Analysis of the Effect of Different Physical Exercise Protocols on Depression in Adults: Systematic Review and Meta-analysis of Randomized Controlled Trials [file sj-docx-1-sph-10.1177_19417381231210286.docx]

**Appendix**

**Table A1.** Characteristics of the included studies

| Study | Participants | Intervention | Outcomes | | PE effect |
| --- | --- | --- | --- | --- | --- |
| Carneiro et al., 2015 [8] | 19 (women) | Aerobic exercise  Moderate-to-vigorous intensity.  Duration 45-50 minutes, 3x/week  16 weeks | | Depression (BDI-II; DASS-21) | Decrease in the parameters of depression. However, there were no differences regarding anthropometry. |
| Forsyth et al., 2015 [18] | 119 (48 women, 26 men) | Individualized program  2x/week  12 weeks. | | Depression and Anxiety (DASS-21) | Both the control group and the intervention group participants in this study improved their mental health, the ingestion of nutrients and the diet quality. |
| McIntyre et al., 2020 [32] | 119 (63 women, 56 men) | Individualized aerobic training program  HR registration. 12 weeks. Afterwards, contacted to encourage the practice. | | Depression (BDI) | Reduced depression and hostility but not anxiety or anger in healthy, sedentary young adults. |
| Soucy et al., 2017 [47] | 39 | Personalized aerobic program.  Gradual increase over 8 weeks.  Duration and frequency registered in a logbook. | | Depression (PHQ-9); Physical activity (LTEQ) | Activation guided self-help interventions, such as PA, are shown to be promising in their ability to reduce the severity of light-to-moderate depression in adults. |
| Szuhany et al., 2019 [48] | 32 (25 women, 7 men) | Completing a total of 150 minutes of moderate-intensity aerobic exercise (600 per month) or light stretching per week.  Autonomy regarding the type of exercise and the weekly schedule.  12 weeks. | | Depression (BDI-II; MADRS); Quality of Life (Q-LES-Q) | In only 9 sessions provided during the program, over 12 weeks, the patients significantly reduced their depression , with 39% of the sample achieving remission. |
| Vanroy et al., 2017 [50] | 135 | Individual walking program with pedometer (duration according to the results of physical fitness). One group walk a week and monitoring.  Registration of the recommended walks and number of steps.  10 weeks. | | Depression (BDI/ Hamilton Depression Rating Scale/ 16-  item self-report Quick Inventory of Depressive Symptomatology)  PA (6-minute Test/ Godin-Shepard Leisure-Time Exercise  Questionnaire) | The current study did not demonstrate any effects of a walking intervention in patients with mental disorder on physical fitness, PA, anxiety levels and depression levels.  However, participants in both conditions increased levels of self-reported PA immediately after the intervention  . This suggests that simply measuring PA levels in the context of a project might be stimulating enough for patients with mental disorders to improve their PA in the short term. |
| Cassandra et al., 2014 [21] | 52 | 5 sessions/week of supervised aerobic exercise.  Warm-up: 5min; 30 minutes of light-to-moderate intensity and, finally, 5 minutes of stretching.  Instructed to perform 3 trainings of 5 minutes with prescribed training intensity at 40-59% of the heart rate reserve (HRR), or between 4-6 according to the perceived exertion scale (RPE).  3 weeks. | | Depression (MADRS) | Encouraging results that aerobic exercise training in addition to pharmacological intervention is effective in reducing depression and in increasing body flexibility among patients with light-to-moderate depression. An early introduction of training in the treatment of depression may have a synergistic effect with pharmacological intervention in order to ensure the effectiveness of the rehabilitation. |
| Danielsson et al., [15] | 42 (32 women, 10 men) | Aerobic exercises  2 individual sessions during 2 weeks.  Later, it was followed by a period of 8 weeks with 2 weekly sessions of 60 minutes/session.  10 weeks. | | Depression (MADRS); Anxiety (BAI); Cardiovascular fitness (VO2max) | This study suggests exercise as an additional therapy, using an individual-centered approach. It has beneficial effects on the severity of depression, as well as on cardiovascular fitness of individuals with severe depression. |
| Chalder et al., 2012 [10] | 361 | Guiding manual with a set of motivational interviewing techniques and behavioral strategies.  Moderate-to-vigorous PA during 150 minutes  a week. | | Depression (BDI-II); Quality of Life; Physical activity | It is clinically effective in the improvement of depression. |
| Saltan et al., 2020 [43] | 64 | Pilates and Therapeutic exercises program  (7 aerobic exercises  and 10 resistance exercises with respective gradual increase).  12 weeks.  Light-to-moderate intensity. | | Depression (BDI) | Both the Pilates approach and the therapeutic exercises may be preferred.  The data from this study can guide the improvement and the practice of similar evidence-based processes to help reduce depression and pain, as well as to increase the quality of life of young adults. |
